# Early outcomes and predictors of patient satisfaction after TKA: a prospective study of 200 cases with a contemporary cemented rotating platform implant design

**DOI:** 10.1186/s40634-021-00347-w

**Published:** 2021-04-17

**Authors:** Corné van Loon, Niels Baas, Verdonna Huey, James Lesko, Geert Meermans, Diederik Vergroesen

**Affiliations:** 1 Orthopaedic Department, Rijnstate Hospital, PO Box 9555, 6800 TA Arnhem, The Netherlands; 2 HAGA Hospital, Sportlaan 600, 2566 MJ The Hague, The Netherlands; 3 Present Address: Bergman Clinics, Laan van Oversteen 20, 2289 CS Rijswijk The Hague, The Netherlands; 4 DePuy Synthes Joint Reconstruction, Inc., PO Box 988, 700 Orthopaedic Drive, Warsaw, IN 46581-0988 USA; 5 Orthopaedic Department, Bravis Hospital, Boerhaaveplein 1, 4624 VT Bergen Op Zoom, The Netherlands; 6 Spaarne Hospital, Spaarne Poort 1, 2134 TM Hoofddorp, The Netherlands

## Abstract

**Purpose:**

The purpose of the study was to identify the earliest time point where subjects realized the greatest clinical improvement after TKA, and the time when post-operative scores became superior to pre-operative scores. Post-hoc exploratory analyses were conducted to investigate predictors of early post-operative outcomes and patient satisfaction.

**Methods:**

Six investigators across 4 sites in the Netherlands prospectively implanted 200 subjects with a contemporary cemented rotating platform device. Patient Reported Outcome Measurements (PROMs) KOOS-PS, PKIP, and EQ-5D were collected pre-operatively and post-operatively through 2-years. PROMs change from pre-operative baseline were summarized, along with radiographic outcomes and adverse events (AEs). Pre-operative patient characteristics were explored for correlation with patient outcomes, and patient satisfaction for correlation with KOOS-PS.

**Results:**

Follow-up compliance was 99% at 6-months, and 95.5% at 2-years. The percentage with higher KOOS-PS compared to baseline was 81.3% at 6-months. KOOS-PS, PKIP, and PKIP subscore means were all better at 6-weeks versus baseline. Gender, BMI, hypertension, and pre-operative KOOS-PS were weakly correlated with 6-week KOOS-PS (multivariate R-squared = 14.1%), but only pre-operative KOOS-PS demonstrated correlation with post-operative KOOS-PS at 6-months or later (R-squared < 5% at 6-months and 2 years). Satisfaction was moderately correlated with concurrent KOOS-PS at each post-operative time point, with (R-squared = 35.3% at 6-months, and 37.5% at 2 years).

**Conclusion:**

The greatest mean clinical improvement occurred within the first 6-weeks. Although some pre-operative factors were correlated with higher early post-operative KOOS-PS outcomes, these advantages disappeared by 6-months aside from weak correlation with pre-operative KOOS-PS. Post-operative KOOS-PS was moderately correlated with concurrent post-operative satisfaction. These results may be used for pre-operative counseling and management of patient’s postoperative expectations.

**Trial registration:**

Clinicaltrials.gov, NCT02339610. Registered 15 January 2015.

## Introduction

Total knee arthroplasty (TKA) has proven to be a successful surgical treatment for reducing pain and improving knee function in patients with arthritis of the knee joint [18]. Although TKA may be deemed a clinical success as assessed by survival analysis and objective clinical outcomes, 1 in 5 patients remain dissatisfied. The most common causes of patient dissatisfaction include residual pain (14%–28%) or limited function (16%–30%) [1, 3, 13] related to poor patient selection, poor patient expectation management, poor surgical technique, and postoperative complications [4, 10, 17, 19].

In an effort to increase patient satisfaction for up to 30% of patients who otherwise have well-functioning implants, numerous studies have been done to determine if a correlation exists between patient preoperative variables and postoperative patient satisfaction. According to Williams et al. [19], the evidence proves to be inconsistent. Williams aligns with earlier research [1, 18] postulating that postoperative factors such as pain and knee function are better predictors of postoperative patient satisfaction than preoperative variables, as early as 3 months postoperative.

The purpose of the study was to follow clinical improvements through 2-years after TKA to identify the earliest clinical time point where subjects realized the greatest improvement, and where post-operative scores become superior to pre-operative scores. Post-hoc exploratory analyses were conducted to investigate predictors of early post-operative outcomes and patient satisfaction. To assess the subject’s perception of their recovery, the Knee Injury and Osteoarthritis Outcome Score Physical Function Short Form (KOOS-PS) was utilized to assess the primary outcome and the Patient’s Knee Implant Performance Score (PKIP) and the EuroQol five dimension 5-level instrument (EQ-5D) change from baseline (CFB) were utilized to assess secondary objectives.

## Materials and methods

This was a prospective, non-randomized, non-comparative, non-controlled study. Investigators implanted the cemented ATTUNE® Knee System, (DePuy Synthes, Warsaw, IN) in either a cruciate retaining rotating platform (CRRP) or posterior stabilized rotating platform (PSRP) configuration, consistent with their standard of care. All devices were implanted with a ligament balancing technique. Additionally, all participating centers were instructed to follow their standard practice regarding surgical process, patellar resurfacing, postoperative anticoagulation and rehabilitation protocol. All implanting investigators were experienced medium to high volume primary TKA surgeons (100 or more procedures annually), and each received didactic and hands on sawbones/cadaver training prior to implanting their first study subject.

From January 2015 through October 2016, 6 investigators across 4 sites in the Netherlands enrolled 208 subjects, 200 of whom had unilateral surgery to receive the implant (115 CRRP, 85 PSRP). Subjects were then followed to the 2-year post-operative visit. This study was registered on www.clinicaltrials.gov on 15 January 2015 under registration number NCT02339610; https://clinicaltrials.gov/ct2/show/NCT02339610?term=NCT02339610.

### Inclusion / exclusion criteria

Subjects were included if they were 22 to 80 years of age, diagnosed with non-inflammatory degenerative joint disease, suitable to receive the implant under study, able to comprehend the study, gave voluntary informed consent, and willing to perform all study procedures and follow-up visits. Subjects were excluded if they had inflammatory arthritis (e.g. rheumatoid arthritis, juvenile rheumatoid arthritis, psoriatic arthritis, systemic lupus erythematosus, etc.), a significant neurological or musculoskeletal disorder or disease that may adversely affect gait or weight bearing (e.g. muscular dystrophy, multiple sclerosis, Charcot disease), were diagnosed and taking prescription medications to treat a muscular disorder that limits mobility due to severe stiffness and pain (such as fibromyalgia or polymyalgia), or were experiencing radicular pain from the spine radiating into the index limb. Subjects were also excluded if they had a contralateral amputation or previous partial knee replacement (unicompartmental, bicompartmental or patellofemoral joint replacement), patellectomy, high tibial osteotomy, or primary TKA in the index knee, had participated in a clinical investigation with an investigational product (drug or device) in the previous 3 months, were pregnant or lactating (females), had less than 3 years of life expectancy, had a psychological disorder that could affect their ability to complete questionnaires or be compliant with follow-up requirements, or were involved in personal injury litigation.

### Data collection and analysis

Data collection included three patient-reported outcome measurements (PROMs): the KOOS-PS (100 point scale), the PKIP (100 point scale; 4 subscores, 10 points each), and the EQ-5D (100 point visual analog scale (VAS), and crosswalk index 0 to 1). The KOOS-PS was chosen as the primary endpoint because it is collected as standard of care in the Netherlands and because it is a relevant subjective measurement to assess recovery [8, 15]. The PKIP was chosen because it was developed specifically to “measure the patient’s perception of their biomechanics; the relationship of function relative to improved stability, motion, satisfaction, and confidence” [6, 7, 11]; and the EQ-5D was selected to assess the Subject’s overall general health status [2, 9].

Subjects were seen pre-operatively for a clinical assessment and to collect medical history, PROMs, and radiographs. Subjects were then required to return to clinic at 6 weeks (1–60 days), 3 months (61–137 days), 6 months (138–303 days), 1 year (304–669 days) and again at 2 years (670–913 days) for clinical, radiographic follow-up and to complete PROMs. The intervals were continuous to accommodate a broad range of standard of care.

The primary endpoint of the study was the KOOS-PS change from baseline (CFB) at 6 months post-op. The primary endpoint analysis was to show that the entire 6 month mean CFB 95% confidence interval (CI) was greater than 0. KOOS-PS, PKIP, and EQ-5D were summarized at each post-operative time-point to assess how quickly patients improved on their path to recovery from the time of surgery through 2-year endpoint after TKA. Pre-operative patient attributes were explored for predictors of early post-operative patient outcomes, and patient satisfaction was explored for possible correlation with KOOS-PS. Standard anteroposterior (AP) and lateral views radiographs were evaluated for femoral (AP) and tibial (AP and lateral) radiolucencies ≥2 mm, osteolysis, subsidence, or signs of aseptic loosening. The types and frequencies of reported serious adverse events (SAEs), device-related and/or procedure-related adverse events are summarized.

### Analysis methodology

The primary endpoint was assessed with a 2-sided 95% CI from a longitudinal model of KOOS-PS CFB over all post-operative timepoints. For the primary endpoint analysis, it was estimated that *N* = 170 would provide 90% power if the true CFB was 7 points, and 99% power if the true CFB was 10 points; it was believed that CFB would be greater than 10 points. The sample size was increased to *N* = 200 for possible attrition. Apart from the primary endpoint analysis, all statistical summaries were basic statistics (means and standard deviations) without the longitudinal model, and change from baseline and comparisons between time-points were assessed with a paired t-test. No data were imputed in cases of missing data. Exploratory analysis for predictors of better KOOS-PS outcomes at various time points was conducted with ordinary least squares multivariate regression. Exploratory analysis for predictors of patient satisfaction at various time points was conducted with ordinal logistic regression, with responses to the following PKIP question 9 as the response variable (PKIP Q9): ‘Overall, how satisfied are you with how your knee functions?’ (response options: ‘Very Dissatisfied’, ‘Dissatisfied’, ‘A little Dissatisfied’, ‘A little Satisfied’, ‘Satisfied’, ‘Very Satisfied’). Although these exploratory regression analyses were not prospectively powered, a sample of *N* = 200 is sufficient to have detected a linear regression R-squared of 4% or higher at an alpha of 0.05 with greater than 80% power.

## Results

Follow-up compliance (KOOS-PS on file) was 99% (198/200) at 6 months, 98.5% (197/200) at 1 year, and 95.5% (191/200) at 2 years. A summary of demographics and operative details is presented in Table 1, and are representative of a typical primary TKA population. No subjects had greater than 20 degrees of varus or valgus malalignment preoperatively.

**Table 1:** Demographics and operative details (mean ± SD or %)

Age	65.4 ± 7.8 years (minimum: 41, maximum: 78; 9 below 50)
Gender	63% female
BMI	29.0 ± 4.3 kg/m2
Primary diagnosis	100% OA
Comorbidities	3% Back pain
	5.5% Cancer
	12% Cardiovascular
	10.5% Diabetes
	7% Endocrine/Metabolic
	6% Gastrointestinal
	17% Hypercholesteremia
	35% Hypertension
	9.5% Respiratory
	5% Vascular
Surgery time (minutes)	56.0 ± 13.1
Patella	24% Resurfaced (42% CRRP, 0% PSRP)
Length of stay (days)	2.8 ± 1.2 days

**Fig. 1:**
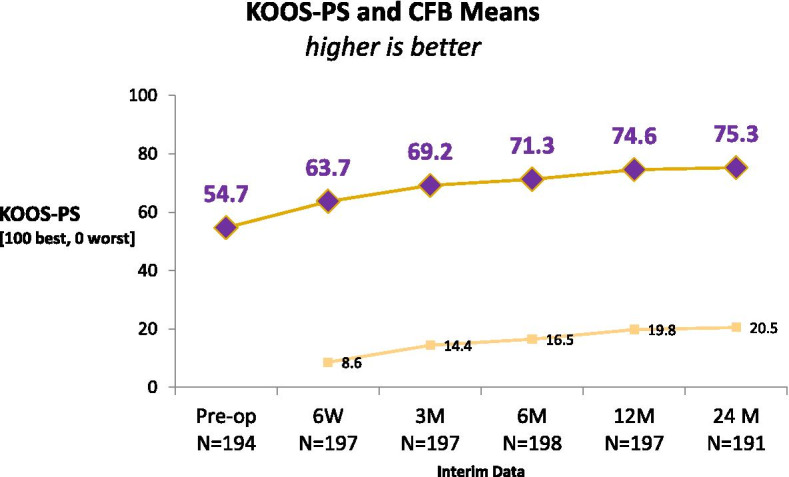
KOOS-PS (top) and CFB (bottom) Means

The 95% CI for the 6-month KOOS-PS CFB from the longitudinal model was (14.8, 18.1), so the primary endpoint analysis was successfully demonstrated. Estimates of PROMs outcomes are provided in Table 2, along with CFB estimates (in italics). As shown in Table 2, means for KOOS-PS, PKIP, and all PKIP subscores were better at 6 weeks compared to baseline (*p*-values < 0.05), and means for EQ-5D crosswalk and VAS were better at 3 months compared to baseline (p-values < 0.05). Differences between 1 and 2 years were not significant for KOOS-PS, PKIP modifying activities or stability subscores, EQ-5D crosswalk or VAS; slight improvements for PKIP Overall, confidence, and satisfaction subscores were noted (*p*-values < 0.05). Of all PKIP subscores, PKIP satisfaction had the greatest increase compared to baseline at each respective post-operative timepoints. KOOS-PS and CFB means are shown in Fig. 1. The percentage of patients with a higher KOOS-PS score compared to baseline was 64.4% (123/191) at 6 weeks, 78.5% (150/191) at 3 months, and 81.3% (156/192) at 6 months. The 2 year KOOS-PS had decreased from 1 year by more than 10 points in 9.9% (19/192) of Subjects; only one had a knee related AE beyond 1 year (arthralgia; not serious).

**Table 2:** PROMs results mean ± SD (CFB in italics)

		Scale	Pre-Op	6 Weeks	3 Month	6 Month	12 Month	24 Month
**KOOS- PS**		0–100	54.7 ± 14.4	63.7 ± 10.2	69.2 ± 11.4	71.3 ± 12.6	74.6 ± 12.5	75.3 ± 13.1
				*8.6 ± 15.3*	*14.4 ± 16.5*	*16.5 ± 16.9*	*19.8 ± 16.6*	*20.5 ± 17.2*
**PKIP**	**Overall**	0–100	33.2 ± 13.3	53.1 ± 13.7	59.1 ± 14.6	62.7 ± 16.7	67.5 ± 17.7	69.9 ± 17.4
				*19.6 ± 16.8*	*25.7 ± 17.9*	*29.3 ± 18.8*	*34.0 ± 20.9*	*36.3 ± 20.4*
	**Confidence**	0–10	4.2 ± 2.0	6.4 ± 1.5	6.7 ± 1.5	7.0 ± 1.7	7.3 ± 1.8	7.7 ± 1.8
				*2.2 ± 2.3*	*2.5 ± 2.2*	*2.7 ± 2.5*	*3.0 ± 2.5*	*3.4 ± 2.5*
	**Stability**	0–10	4.0 ± 2.1	6.4 ± 1.7	6.7 ± 1.6	6.9 ± 1.8	7.5 ± 1.9	7.7 ± 1.7
				*2.5 ± 2.4*	*2.8 ± 2.3*	*3.0 ± 2.5*	*3.5 ± 2.5*	*3.7 ± 2.4*
	**Satisfaction**	0–10	2.7 ± 1.5	6.4 ± 2.0	6.7 ± 1.9	7.1 ± 2.0	7.5 ± 1.9	7.8 ± 1.8
				*3.7 ± 2.3*	*4.0 ± 2.3*	*4.4 ± 2.3*	*4.8 ± 2.5*	*5.1 ± 2.3*
	**Modifying activities**	0–10	4.3 ± 2.5	4.7 ± 2.6	5.7 ± 2.6	5.8 ± 3.0	6.4 ± 3.0	6.5 ± 3.0
				*0.3 ± 3.5*	*1.4 ± 3.4*	*1.5 ± 3.7*	*2.1 ± 3.8*	*2.2 ± 3.7*
**EQ5D**	**Crosswalk**	0 to 1	0.61 ± 0.18		0.80 ± 0.12	0.80 ± 0.13	0.84 ± 0.13	0.86 ± 0.14
					*0.19 ± 0.18*	*0.19 ± 0.20*	*0.23 ± 0.20*	*0.25 ± 0.20*
	**VAS**	0–100	72.3 ± 17.6		79.7 ± 14.5	81.3 ± 14.2	82.6 ± 15.6	82.1 ± 14.1
					*7.7 ± 18.9*	*9.0 ± 18.1*	*10.3 ± 18.6*	*10.0 ± 19.2*

### Exploratory analysis: predictors of early post-operative outcomes and patient satisfaction

Table 3 shows pre-operative patient characteristics that demonstrated correlation with better KOOS-PS in respective multivariate regression models at various time points (*p*-values in parenthesis); a separate regression model was fit for each respective time point. As can be seen by the relatively low coefficient of variation (R-squared) values in these models, the correlation of pre-operative patient characteristics with post-operative KOOS-PS was fairly weak; only pre-operative KOOS-PS was correlated with post-operative KOOS-PS at 6 months or later.

**Table 3:** Regression models of post-operative KOOS-PS vs. pre-operative patient characteristics

KOOS-PS Timepoint	Significant predictor variables* (***p***-values)	R-squared
6 Week	Pre-op KOOS-PS (0.007), History of hypertension (0.005), Gender** (0.033), *BMI* (0.006)	14.1%
3 Month	Pre-op KOOS-PS (0.041), History of hypertension (0.039), Gender** (0.007)	8.6%
6 Month	Pre-op KOOS-PS (0.002)	4.8%
1 Year	Pre-op KOOS-PS (0.001)	6.1%
2 Year	Pre-op KOOS-PS (0.003)	4.7%

*Italics indicates negative correlation

**Men showed slightly higher KOOS-PS at 6 Weeks and 3 Months

Ordinal logistic regression models of PKIP Q9 showed little correlation with pre-operative patient characteristics (separate univariate models were fit for each respective time point); age was weakly correlated with PKIP Q9 at 6 weeks and 6 months, with *p*-values (Cox and Snell R-squared) of 0.018 (2.7%) and 0.020 (2.8%), respectively; there was slightly higher satisfaction with increased age at these timepoints. Under separate univariate logistic regression models for each respective time point of PKIP Q9 was highly correlated with concurrent KOOS-PS, with p-values < 0.001 at each time point, and R-squared values (Cox and Snell) of 24.9%, 28.0%, 35.3%, 41.8%, and 37.5% at 6 weeks, 3 months, 6 months, 1 year, and 2 years, respectively; all showed that the higher the KOOS-PS outcome, the higher the satisfaction.

### Radiographic outcomes

There were no femoral radiolucencies ≥2 mm or findings of subsidence or osteolysis on lateral radiographs at any post-operative time-point, and there were no tibial radiolucencies ≥2 mm or findings of subsidence or osteolysis on either AP or lateral radiographs. One Subject had a finding of tibial aseptic loosening on a 3 month AP radiograph, which was not seen in subsequent radiographs.

### Adverse events

There were no reported intraoperative complications. SAEs were reported in 23% (46/200) of Subjects, including 10 SAEs in 8 Subjects related to either the device or procedure: 1 joint lock (surgical intervention to remove cement), 2 wound drainage and 1 infection in 1 Subject (liner exchange), 1 wound drainage (in a different subject; I&D with no components removed), 3 joint stiffness (manipulation under anaesthesia in all 3), 1 deep vein thrombosis, and 1 haematoma (I&D with no components removed). There was one revision in the study at 31 days (insert exchange for infection as noted above).

## Discussion

The most important finding of the present study was 81.3% (approximately 4 out of 5) of Subjects had a higher 6-month KOOS-PS score than baseline, with the greatest clinical improvement occurring within the first 6 weeks. If we examine means, all 6 week PROMs means were higher than baseline, indicating that the population shifted toward better outcomes by 6 weeks post-operatively. Further, even beyond the 6-month timepoint, all PROMs means continued to improve up to the 2 year follow up.

Liddle [12] compared 6 months PROMs data between TKA and unilateral knee arthroplasty (UKA) and found favourable results in UKA, such as an EQ-5D mean score of 0.772 as compared to the TKA EQ-5D mean score of 0.80 in this study. Also, these study results are comparable with Mathijssen [14] describing Persona® TKA (Zimmer, Warsaw Indiana) with 2 year follow up using KOOS-PS. However, specific to this study, the use of the EQ-5D highlighted the study subject’s expressed well-being and the PKIP scores were reflective of the subject’s perception of knee function in relation to the implant’s stability and motion, the subject’s confidence with natural motion such as ascending and descending stairs, and the subject’s satisfaction with the ability to return to normal activities pain free [11]. In the present study, the PKIP satisfaction subscore increased the greatest during follow up, especially the first 6 weeks, more than the PKIP confidence, stability, and modifying activities subscores. These good results in the first 6 weeks may be due to some of the unique landmark features of the RP system in this current study, with or without resurfacing: the gradually reducing radius in the geometry of the femoral component and varying femoral gutter angle in different femur sizes by more closely mimicking the anatomical patellofemoral joint and facilitating more natural femoral rollback during flexion, even without patella resurfacing [5, 16].

In the early post-operative phase prior to 6 months, KOOS-PS outcomes were only weakly correlated with pre-operative factors: gender (males were slightly higher), BMI (higher BMI was slightly lower), history of hypertension (those with a history were slightly higher), and pre-operative KOOS-PS (those with higher pre-operative KOOS-PS were slightly higher post-operative). By 6 months, there was no noticeable correlation between pre-operative factors and KOOS-PS outcome aside from a very weak correlation with pre-operative KOOS-PS (those with higher pre-operative KOOS-PS were slightly higher post-operative). We interpret this to mean pre-operative patient profile may give a minor advantage to some patients, depending on gender, BMI, history of hypertension, and pre-operative clinical condition, but this minor advantage does not persist beyond 6 months aside from a very weak correlation with pre-operative clinical condition: those doing better pre-operatively may have a very slight advantage for better clinical outcome beyond 6 months. This message to patients may help them know what to expect post-operatively, particularly prior to 6 months.

Post-operative satisfaction showed little correlation with pre-operative patient characteristics, aside from age, which was weakly correlated with slightly higher satisfaction at 6 weeks and 6 months but not beyond. Post-operative satisfaction was moderately correlated with concurrent KOOS-PS at all respective timepoints; those with higher KOOS-PS were more satisfied.

There were several limitations in this study. The study was non-randomized with no control group, and surgeons chose their own preferred configuration of the TKA design (CRRP or PSRP) and whether or not to resurface the patella, possibly introducing selection bias. There were only four sites, so the spectrum of differences between hospitals, surgeons, and post-operative protocols may not have been fully represented in the study.

However, the present study has unique data of 200 cases with 99% complete data at 6 months follow-up and 95.5% follow-up at 2 years. Furthermore, there were 3 postoperative measuring points within 6 months, allowing analysis of improvement between shorter intervals.

## Conclusion

The study demonstrated that 81.3% of patients in this study had a higher 6-month KOOS-PS score than pre-operative baseline; on average, the greatest clinical improvement occurred within the first 6 weeks. Although there may be pre-operative factors that give a slight advantage to early post-operative KOOS-PS outcomes, these advantages disappear by 6 months aside from a weak correlation with pre-operative KOOS-PS condition. There is no particular pre-operative factor that is highly correlated with post-operative satisfaction; post-operative KOOS-PS is moderately correlated with post-operative satisfaction. These results may be useful for pre-operative counseling and management of patient’s postoperative expectations.

## Data Availability

Datasets have not been shared publicly, but may be requested through the Yale Open Data Access program.

## Abbreviations

AEs: Adverse events
AP: Anteroposterior
CFB: Change from baseline
CI: Confidence interval
CRRP: Cruciate retaining rotating platform
EQ-5D-5L: EuroQol five dimension, 5-level evaluation
KOOS-PS: Knee Injury and Osteoarthritis Outcome Score Physical Function Short Form
PKIP: Patient’s Knee Implant Performance evaluation
PROMs: Patient reported outcome measurements
PSRP: Posterior stabilized rotating platform
SAEs: Serious adverse events
TKA: Total knee arthroplasty
UKA: Unilateral knee arthroplasty
VAS: Visual analog scale
